# Redspotted Grouper Nervous Necrosis Virus and the Reassortant RGNNV/SJNNV In Vitro Susceptibility against a Commercial Peroxy-Acid Biocide under Different Conditions of Use

**DOI:** 10.3390/vetsci10020076

**Published:** 2023-01-19

**Authors:** Enrico Volpe, Francesca Errani, Samuele Zamparo, Sara Ciulli

**Affiliations:** 1 Department of Veterinary Medical Sciences, Alma Mater Studiorum, University of Bologna, 47042 Cesenatico, FC, Italy; 2 Azienda Agricola Troticoltura Erede Rossi Silvio di Rossi Niccola, via Madonna dei Calcinai 2, 62025 Sefro, MC, Italy

**Keywords:** disinfectant, virucidal activity, biosecurity, betanodavirus, viral nervous necrosis, reassortant RGNNV/SJNNV, Redspotted grouper nervous necrosis virus

## Abstract

**Simple Summary:**

Redspotted grouper nervous necrosis virus (RGNNV) and its reassortant strains belonging to the genus *Betanodavirus* represent the most threatening viral pathogens in the Mediterranean aquaculture sector. Due to the sector’s ongoing expansion, controlling the spread of viral encephalopathy and retinopathy (VER) caused by betanodaviruses is critical. For this reason, implementing strict hygienic standard procedures primarily based on disinfection could significantly reduce the spread of VER. In this study, two standardized European protocols (BS EN 14675:2015 and BS EN 17111:2018) were applied in order to assess the virucidal activity of a commercial peroxy-acid biocide against the RGNNV and its reassortant RGNNV/SJNNV strain. The disinfectant was effective in reducing the viral titer, but with different results depending on concentrations, strains, and conditions of application.

**Abstract:**

Aquaculture is a constantly growing sector. The intensification of fish production and the movement of aquatic animals could cause the spread of infectious diseases. Remarkably, the diffusion of viral agents represents the major bottleneck for finfish production, and viral encephalopathy and retinopathy (VER) is considered the most impacting disease for Mediterranean aquaculture. No effective therapies are available to contrast VER, and vaccination can be applied only in grow-out facilities. Hence, programs to minimize the sanitary risks in farms are paramount to implementing hygienic standards and biosecurity. This study aimed to evaluate the in vitro virucidal activity of a peroxy-acid disinfectant (Virkon^®^ S, DuPont, Sudbury, UK) towards the two NNV strains most widespread in the Mediterranean Sea. Remarkably, two protocols were applied to assess the virucidal activity under different conditions of use: the suspension test and the net test. The latter has been applied to evaluate the efficacy of the biocide on instruments, simulating the in-field application. The obtained results demonstrated the suitability of the tested biocide for NNV inactivation, being effective under some of the tested conditions. However, the presence of organic matter, the concentration of the product, and the application conditions can significantly affect the result of the disinfection procedure.

## 1. Introduction

In recent years the worldwide aquaculture sector has been experiencing a constant and continuous increase in production with a consequent growth in intensive fish farming [1]. This intensification of fish production requires implementing hygienic standards to minimize sanitary risks due to the diffusion of infectious diseases. In fact, among the most significant challenges that aquaculture has to face are viral diseases [2]. Particularly, viral encephalopathy and retinopathy (VER) represent the most impacting disease for Mediterranean aquaculture [3,4,5]. VER is caused by the nervous necrosis virus (NNV), a small non-enveloped RNA virus highly resistant in the aquatic environment, and is included in the Nodaviridae family, Betanodavirus genus [6]. Betanodavirus contains a bisegmented genome composed of two single-stranded positive-sense RNA molecules [7]. The sequence of RNA1 is about 3.1 kb encoding a RNA-dependent RNA polymerase (RdRp) [8], whereas the sequence of RNA2 (1.4 kb) encodes the capsid protein [5]. Based on the analysis of the viral genome, betanodaviruses have been clustered into four species: striped jack nervous necrosis virus (SJNNV), tiger puffer nervous necrosis virus (TPNNV), barfin flounder nervous necrosis virus (BFNNV), and redspotted grouper nervous necrosis virus (RGNNV) [6,9]. Among these, the most widespread in the Mediterranean basin is the RGNNV, which affects mainly the European sea bass (*Dicentrarchus labrax*) farms and could cause high economic losses [3]. However, the emergence of the RGNNV/SJNNV reassortant strain derived from the reassortment between RGNNV and SJNNV genotypes also caused high mortality outbreaks in gilthead sea bream (*Sparus aurata*) larvae [4,10]. Moreover, NNV has also been detected in numerous wild marine fish species and invertebrates in the Mediterranean Sea [11,12,13]. Notably, NNV-contaminated bivalve mollusks can release infectious viral particles into waters and the surrounding environment, representing a viral source [14]. Hence, Mediterranean aquaculture facilities are constantly exposed to the risk of introducing NNV from contaminated water and the surrounding environment.

Currently, no effective therapies are available for VER, and vaccination in Mediterranean aquaculture is limited to grow-out facilities. Therefore, the control in the hatcheries, where the mortality rates reach the highest values, is based on direct prophylaxis to prevent the virus from entering and spreading in the farms. For this purpose, implementing strict hygienic standard procedures could significantly reduce the spread of VER [2,3].

One valuable tool already available for VER control is the early detection of viral genome presence thanks to PCR-based methods already developed [15,16]. However, in order to limit or prevent the viral spread and to efficiently manage the outbreaks, identifying effective biocides against NNV is of paramount importance [2].

To undertake adequate control plans, in-depth knowledge of the characteristics of the pathogen’s resistance and the availability of effective disinfection products and protocols in the various stages of the production cycle is essential [17]. Previous studies have tested the resistance of some fish viruses against physical and chemical agents to increase knowledge of pathogens’ resistance and establish appropriate control methods [18,19,20,21,22].

These studies pointed out the NNV resistance in a wide range of environmental conditions for an extended period. Furthermore, chlorine and iodine-based disinfectants have proven to be highly effective, but only in the absence of organic substances [19], showing how different test conditions could affect biocide effectiveness. Nevertheless, most of the studies dealing with the evaluation of biocides’ virucidal activity do not apply standardized methods, providing results that are barely comparable. To standardize the assessment of biocide effectiveness, normative documents are available and their application is recommended to ensure that users have effective, high-quality products at their disposal [23].

The present study aims to assess a commercial disinfectant’s (Virkon^®^ S, DuPont, Sudbury, UK) in vitro virucidal activity towards two different NNV strains (RGNNV and RGNNV/SJNNV) to date, representing the two NNV variants most spread in the Mediterranean Sea. The virucidal activity was assessed under two protocols based on European standards: the suspension virucidal activity test and the net virucidal activity test, to simulate the in-field conditions and establish the effectiveness of the disinfectant for use on instruments.

## 2. Materials and Methods

### 2.1. Viruses

In this study, two previously characterized NNV strains were used for the in vitro virucidal activity tests: the RGNNV strain It/351/Sb, isolated from *D. labrax* [24]; and the reassortant RGNNV/SJNNV strain Sa-416-Dec17, isolated from *S. aurata* [10] were propagated in a striped snakehead cell line (SSN-1) and titrated on 96-well plates. The titer was expressed as the viral dilution infecting 50% of cell cultures (TCID_50_ mL^−1^). TCID_50_ was calculated according to the Spearman–Karber method [25].

### 2.2. Suspension Virucidal Activity Test

The protocol used to evaluate the virucidal activity of a commercial peroxy-acid compound (Virkon^®^ S, DuPont, Sudbury, UK) against two different NNV strains, the RGNNV and the reassortant RGNNV/SJNNV, was based on the European BS EN 14675:2015 [26] standard.

The protocol has the purpose of evaluating the virucidal activity of chemical disinfectants and antiseptics used in the veterinary area through a quantitative suspension test. According to Verner-Jeffrey and colleagues, the procedure was adapted to test aquatic pathogens [20]. Starting virus concentrations were 10^8.2^ TCID_50_ mL^−1^ and 10^6.6^ TCID_50_ mL^−1^ for the RGNNV strain, It/351/Sb, and the reassortant RGNNV/SJNNV strain, Sa-416-Dec17, respectively.

Before performing the virucidal activity test, the cytotoxicity assays of the commercial peroxy-acid compound were carried out according to the European BS EN 14675:2015 standard to evaluate the actual toxicity of the disinfectant on cells (cytotoxicity test A) and the reduction in the cells’ sensitivity to the virus exposed to the disinfectant (cytotoxicity test B). Cytotoxicity A was estimated by exposing the cells’ monolayers to 10-fold dilutions of the disinfectant using 1 and 2% *v*/*w* starting concentrations and checking the cells’ disruption after 1 h of incubation. Cytotoxicity B was evaluated by comparing a virus titration conducted on a cell monolayer that was treated with the disinfectant prior to the viral exposure with a titration performed on a cell monolayer treated with culture medium.

Then the virucidal activity was assessed with an exposure time of 5 min at a temperature of 20 °C. The effect of interfering substances was also tested as described in the protocol, as all the experiments have been conducted in low-soiling level (3 g L^−1^ bovine albumin solution) and high-soiling level (10 g L^−1^ bovine albumin solution and 10 g L^−1^ yeast extract) for comparison. Sea water (SW) was used as a diluent. Two disinfectant concentrations were tested: 0.5 and 1% *w*/*v*.

The suspension virucidal activity test was carried out through comparative viral titration between a solution containing 80% disinfectant, 10% interfering substance, and 10% viral suspension, and a solution in which SW replaced the disinfectant as a negative control. The solutions were 10-fold diluted and titrated on 96-well plates of SSN-1 cells. Plates were incubated at 25 ± 1 °C for seven days. All the experiments were conducted in triplicate.

According to the European BS EN 14675:2015 standard, the virucidal activity of the product was expressed as titer reduction (TR) calculated by subtracting the logarithmic titer of the disinfectant dilution from the logarithmic titer of the virus control. When no cytopathic effect was observed, a value of 10^2.5^ TCID_50_ mL^−1^ was assigned corresponding to the lower viral titer detectable, and the TR was expressed with the lower virucidal value prefixed with “≥” as requested by the European BS EN 14675:2015 standard. TR values are defined as the mean of the three repeats ± standard deviation (SD).

### 2.3. Net Virucidal Activity Test

In order to establish whether the biocide for use on instruments (nets, cages, etc.) has virucidal activity in the field, a new protocol (net test) simulating in-field application, was developed. The protocol was based on the European Standard BS EN 17111:2018 [27], which aims to create a quantitative carrier test to evaluate the virucidal activity on instruments used in the medical area. Furthermore, the procedure was adapted against the two NNV strains (RGNNV and the reassortant RGNNV/SJNNV), already subjected to the suspension virucidal activity test.

A nylon net of 1 cm^2^ was cut and immersed in a 1 mL solution containing 90% of the viral solution (with a titer of 10^8.2^ TCID_50_ mL^−1^ and 10^6.6^ TCID_50_ mL^−1^ for RGNNV and RGNNV/SJNNV, respectively) and 10% of the interfering solution. Then, the net was air-dried for 30 min and immersed in 1 mL of disinfectant solution or SW (negative control) to simulate procedures usually conducted at farm level.

The contact time with the disinfectant, temperature of incubation, biocide concentrations, and soiling conditions was the same as those used in the suspension virucidal activity test.

After the immersion with the disinfectant, to avoid a cytotoxic effect of the first dilutions of the tested biocide, the nylon net was immersed in a 1 mL solution of Leibovitz’s L-15 Medium supplemented with 2% *v*/*v* faetal bovine serum (FBS) and 10% *w*/*v* sodium thiosulfate. 

The plate incubations and TCID_50_ calculations were carried out as reported in Section 2.2. When no cytopathic effect was observed, a value of 10^1.5^ TCID_50_ mL^−1^ was assigned corresponding to the lower viral titer detectable, and the TR was expressed with the lower virucidal value prefixed with “≥” as requested by the European BS EN 17111:2018 standard. TR values are defined as the mean of the three repeats ± standard deviation (SD).

### 2.4. Statistical Analyses 

Experiments were conducted in triplicate for each exposure concentration of the disinfectant and soiling interference. Statistical analyses were performed using the statistical software GraphPad Software (San Diego, CA, USA), in which data were subjected to a two-way ANOVA using disinfectant concentration and soiling level as variables. The level of statistical significance acceptance was *p* < 0.05.

## 3. Results

### 3.1. Suspension Virucidal Activity Test

The European BS EN 14675:2015 standard protocol was successfully applied to assess the virucidal activity of the tested commercial peroxy-acid compound against the two NNV strains. Cytotoxicity was efficiently reduced through dilution and chemical neutralization (using sodium thiosulphate), according to the European BS EN 14675:2015 standard and as already reported by Verner-Jeffreys et al. [20] for chemicals of the same class. In particular, the cytotoxicity test A results allowed the evaluation of disinfectant activity starting from the second 10-fold serial dilution (10^−2^) which corresponded to a minimum value of 10^2.5^ TCID_50_ mL^−1^. Cytotoxicity test B, conducted with the highest but non-toxic concentration of the disinfectant, showed a reduction in the viral titer of 0.2 log TCID_50_ mL^−1^ compared to the control titration. This value is in accordance with the European BS EN 14675:2015 standard that requests a difference of <1 log between viral titration obtained from cells treated with and without the disinfectant.

The suspension virucidal activity test demonstrated a similar virucidal effect for the commercial peroxy-acid disinfectant, Virkon^®^ S, against the two tested NNV strains (RGNNV genotype and the RGNNV/SJNNV reassortant strain). 

**Figure 1:**
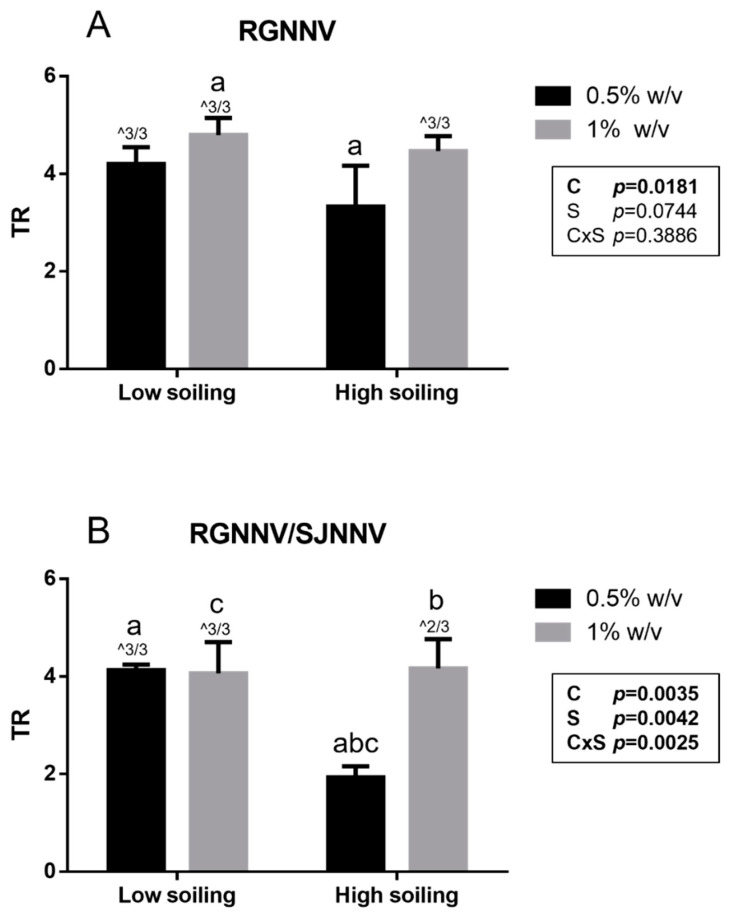
Suspension test results obtained for the RGNNV strain (**A**) and the reassortant RGNNV/SJNNV strain (**B**). TR: titer reduction; C: disinfectant concentration; S: soiling level. The same superscript letters indicate significant differences among titer reduction values (*p* ≤ 0.05). ^ indicates the number of test repeats where no CPE was detected.

Notably, a titer reduction (TR) ≥ 4 log, considered effective by the BS EN 14675:2015 standard, was demonstrated for the tested biocide at a concentration of 1% *w*/*v* under both low- and high-soiling conditions (Figure 1). Similarly, a TR ≥ 4 log was observed for the tested biocide at the concentration of 0.5% *w*/*v* towards both strains when tested under low-soiling conditions. However, when tested under high-soiling conditions at the 0.5% *w*/*v* concentration, the biocide showed a significantly higher titer reduction (*p* < 0.05) towards the strain It/351/Sb (TR 3.3 ± 0.8 log) than the titer reduction in the Sa-416-Dec17 strain (TR 1.9 ± 0.2 log). As a matter of fact, in this condition, no effective TR was reached (≤4 log). Furthermore, statistical analysis showed that for the RGNNV strain, TR was significantly affected by disinfectant concentration (*p* = 0.0181), whereas for RGNNV/SJNNV, strain TR was influenced by both disinfectant concentration (*p* = 0.0035) and soiling level (*p* = 0.0042) with an interaction between them (*p* = 0.0025; Figure 1). 

### 3.2. Net Virucidal Activity Test

**Figure 2:**
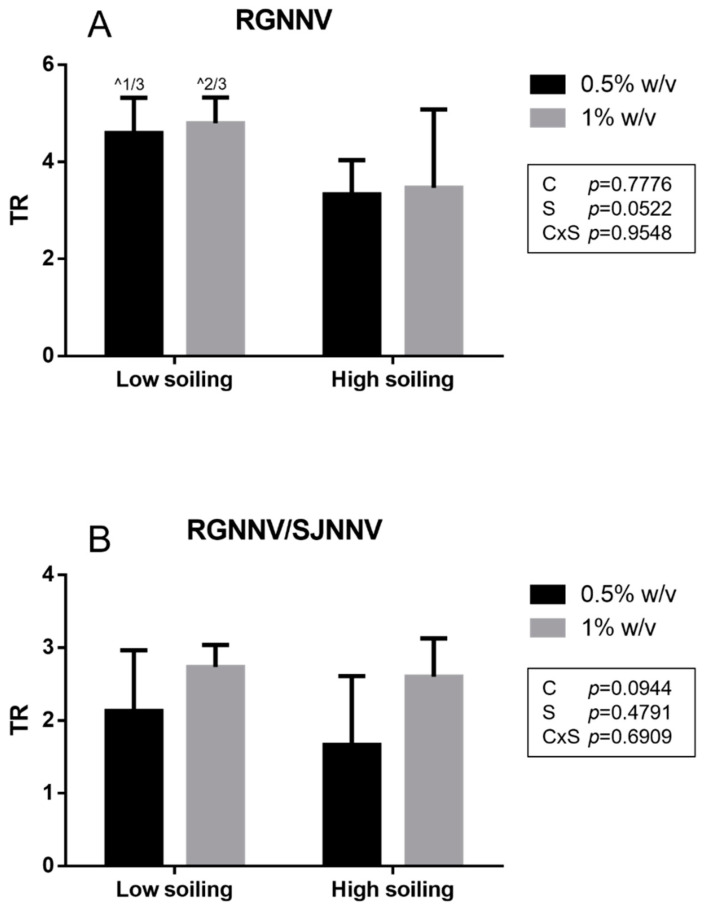
Net test results obtained for the RGNNV strain (**A**) and the reassortant RGNNV/SJNNV strain (**B**). TR: titer reduction; C: disinfectant concentration; S: soiling level. The same superscript letters indicate significant differences among titer reduction values (*p* ≤ 0.05). ^ indicates the number of test repeats where no CPE was detected.

In the net virucidal activity test, a titer reduction (TR) ≥ 4 log was obtained for the tested biocide at the concentration of both 0.5 and 1% *w*/*v* only under low-soiling conditions for It/351/Sb viral strain (RGNNV). A slightly lower TR was observed under high-soiling conditions (TR 3.3 ± 0.7 log and 3.5 ± 1.6 log at 0.5 and 1% *w*/*v*, respectively) for the It/351/Sb strain (Figure 2).

Similar to the suspension test, a significantly higher titer reduction (*p* < 0.05) towards strain It/351/Sb (TR 3.3 ± 0.7 log and 3.5 ± 1.6 log at 0.5 and 1% *w*/*v*, respectively) compared to the titer reduction in the Sa-416-Dec17 strain (TR 1.7 ± 0.9 log and 2.6 ± 0.5 log at 0.5 and 1% *w*/*v*, respectively) was observed when tested under low-soiling conditions at both 0.5 and 1% *w*/*v* concentrations. For the RGNNV/SJNNV reassortant strain Sa-416-Dec17, all TR values were <4 log for all tested conditions.

Furthermore, the net test allowed us to estimate the titer reduction due to air drying. Comparison between viral titers before (10^8.2^ TCID_50_ mL^−1^ and 10^6.6^ TCID_50_ mL^−1^ for RGNNV and RGNNV/SJNNV, respectively) and after (10^6.8±0.2^ TCID_50_ mL^−1^ and 10^5.1±0.4^ TCID_50_ mL^−1^ for RGNNV and RGNNV/SJNNV, respectively) the air-drying step showed a reduction of 1.4 and 1.5 log for RGNNV and RGNNV/SJNNV strains, respectively.

## 4. Discussion

The intensification of fish production requires farming techniques with particular attention to hygienic-sanitary aspects aimed at limiting the damages derived from infectious diseases. Regarding Mediterranean aquaculture, viral encephalopathy and retinopathy (VER) is the most important viral disease responsible for economic losses in the Mediterranean basin [2], affecting European sea bass and gilthead sea bream farms. Currently, there are no available therapeutic aids able to limit the damages caused by this disease, and vaccines can be applied only in the grow-out stage of production; hence, direct prophylaxis remains the best tool to reduce the frequency of this disease or even avoid it.

Disinfection is a process for inactivating, killing, or destroying pathogenic microorganisms and represents one of the procedures primarily included in biosecurity programs. Many disinfecting practices are based on chemical or physical (heat, ultraviolet light, ultrasonic waves, or radiation) methods. Disinfection in aquaculture represents one pillar of pathogen inactivation; however, the utilization of each disinfectant depends upon its suitable concentration and contact time to eliminate pathogens in fish hatcheries and grow-out facilities [17]. Several factors, such as contact time, the presence of organic material, and temperature are known to affect the effectiveness of a disinfectant [2]; however, limited data about disinfection procedures’ effectiveness are available about in-field application [28].

This study has focused on in vitro trials aimed at the evaluation of the specific virucidal activity of a commercial product named Virkon^®^ S, to increase knowledge about its effectiveness against betanodaviruses.

The biocide tested in this study, through the suspension virucidal activity test, resulted in being effective against RGNNV and RGNNV/SJNNV at the manufacturer’s recommended concentration of 1% at both high- and low-soiling levels. The broad spectrum of Virkon^®^ S against fish viruses has already been tested: 0.5% Virkon^®^ S revealed a strong virucidal effect against salmonid alphavirus, inducing >4 log TCID_50_ mL^−1^ reductions after 5 min contact time under high soiling conditions at 4 °C [21]. Furthermore, immersion of a fishing net in 0.5 and 1% Virkon^®^ S solutions for 2 or 5 min lessens infectious pancreatic nervous virus (IPNV) titer at ≥4 log reductions at 15 °C [28]. The sensitivity of NNV to a broad spectrum of physicochemical disinfectants was tested by Frerichs and colleagues [19] several years ago, but the NNV genotype used was not reported. Furthermore, to our knowledge, no data are available on the newly emerged RGNNV/SJNNV reassortant strain susceptibility to disinfectants.

Regarding the net test, a TR ≥ 4 log TCID_50_ mL^−1^, corresponding to the reduction of 99.99% of virus particles, was obtained only for the RGNNV strain, showing how different conditions can affect the effectiveness of the disinfection process.

Even if only a few data are available, in order to better understand the differences in disinfectant effectiveness between the RGNNV and RGNNV/SJNNV strains used in this study, information about disinfectant mode of action and viral strain characteristics is discussed. Betanodaviruses are small, non-enveloped viruses; considering the viral particle structure, non-enveloped viruses are much more resistant to chemical disinfectants and antiseptics than enveloped viruses [23]. Moreover, within the non-enveloped virus group, the so-called large viruses are more susceptible to disinfection than small viruses; the most effective disinfectants to inactivate this highly resistant group of viruses are strong oxidizing agents such as hydrogen peroxide and peracetic acid [29]. Virkon^®^ S is composed of a mixture of a potent oxidizing agent (potassium peroxymonosulfate triple salt 40–55%) and a surfactant (sodium toluenesulphonate). Peroxymonosulfate inactivates microorganisms through a broad spectrum, non-selective range of oxidation reactions including the generation of hydroxyl radicals which can subsequently oxidize proteins [30,31]. Accordingly, the assumed mechanism of NNV inactivation may be based on coat-protein oxidation.

Despite the fact that the two tested strains have the same structure, differences have been reported at the coat-protein level. The RGNNV/SJNNV reassortant strain genome is composed of RGNNV-type RNA1 and SJNNV-type RNA2 genome segments acquiring properties of SJNNV coat protein. The Betanodavirus RNA2 genome segment, which encodes for the coat protein, is characterized by a conserved and a variable region. The nucleotide similarity between the RGNNV and SJNNV RNA2 segments is 77.8%, with a maximum value of 96.3% for the conserved region and a minimum value of 63.0% for the variable region. These genome differences are reflected in the 82.1% similarity of amino acid sequences. Furthermore, the RGNNV RNA2 genome segment lacks six bases resulting in a deletion of two amino acids compared to the coat protein coded by the SJNNV genotype [32]. On the other hand, the RGNNV parental strain and the RGNNV/SJNNV reassortant strain on the basis of the RNA1 genome segment belong to the same phylogenetic clade [33].

Overall, these viral features influence important biological characteristics, such as host range, pathogenicity, and antigenic properties [10,33,34]. Recently, NNV coat-protein structure prediction showed that amino acid substitutions could play a role in affecting the hydrophobicity varying in the SJNNV, RGNNV, and reassortant strains [35]. This study focuses on the relationship between amino acid sequence charge and strain virulence; however, a change in the amino acid chemical characteristics could also affect the disinfectant’s effectiveness against the two tested viral strains.

Furthermore, the net test shows that the air-drying step produces a viral titer reduction of about 1.5 log TCID_50_ mL^−1^ for both the analyzed strains, highlighting the importance of adding an air-drying step in the disinfection procedures. Protocols including carrier tests (plastic, glass, or metal carriers) have been already used to test the effect of drying on viruses [22,36,37]. In accordance with their higher resistance to chemical compounds, non-enveloped viruses are also more resistant to drying compared to enveloped viruses. Interestingly, the tested NNV strains showed a higher susceptibility to the drying procedure compared to other non-enveloped viruses previously tested, reaching a titer reduction of 1.5 log TCID_50_ mL^−1^ [37]. Accordingly, a previous study reported an NNV inactivation of 90% after 24 h of drying at room temperature [22].

Studies dealing with disinfectant effectiveness in the aquaculture sector lack a standardized way of analysis. For this reason, in the present study, two standard protocols already available across the European Union were applied with slight changes to be adapted to aquatic animals’ viruses. In particular, while standardized disinfectant testing is based on 30 min exposure time (BS EN 14675:2015 standard), here, we use exposure times of 5 min, representing what we consider a more realistic in-field disinfection duration. In the field, the contact time can be further extended when nets are left wet with a disinfectant following dipping; however, a 5 min time is likely to reflect a contact time more likely to be seen, especially when nets are rinsed in clean water immediately following dipping [28]. Moreover, the standard protocol BS EN 17111:2018, previously applied to the medical sector, has been successfully applied to the aquaculture sector, adding a valuable tool able to assess the disinfectant’s virucidal activity when applied to surfaces or instruments. Furthermore, the neutralization dilution method recommended by BS EN 14675:2015 resulted in being suitable for the neutralization of the biocide tested as already reported for other compounds belonging to the same class [20]. In the net test, in order to increase the neutralization effect, sodium thiosulphate was added as a chemical neutralizer in addition to the neutralization dilution method, increasing the sensitivity of the test. The application of chemical substances to neutralize biocide effects has been widely applied in several studies dealing with pathogens [38,39,40,41].

One more critical point of paramount importance in disinfection procedures is the presence of organic matter on tools or surfaces subjected to disinfection. The soil level affected the disinfectant’s effectiveness against the two tested NNV strains, highlighting the importance of adding a cleaning step before disinfection. In particular, high-soiling conditions lessened the titer reduction under the effective level (TR ≥ 4 log TCID_50_ mL^−1^) in the suspension test when disinfection was conducted at a 0.5% *w*/*v* concentration against both viral strains. Moreover, the disinfection of surfaces was even more critical as shown by the application of the net test. In this case, only low-soiling conditions permitted to effectively affect the titer of the RGNNV strain. In this respect, an air-drying phase could significantly reduce the viral titer improving the effectiveness of the disinfection. Due to the demand for optimization of disinfection protocols to achieve the best results in terms of effectiveness and time, but also from an economic point of view, it is important to explore the actual effectiveness of low-concentration conditions. In this regard, the results of the present study can contribute by showing how to enhance the efficacy of the biocide at the lowest concentration tested.

Finally, the results obtained in this study led to an increase in the knowledge of NNV disinfectant sensitivity, in particular for the RGNNV/SJNNV reassortant strain. The assessed disinfection procedures represent prophylaxis measures suitable for routine application during production in order to prevent virus spread and disease outbreaks.

## 5. Conclusions

To support aquaculture sector growth, it is crucial to develop effective disinfection protocols suitable for field application, and able to counteract infectious diseases limiting the spread of pathogens. The application of normative documents can guarantee effective and high-quality products to users. Two European standardized protocols were successfully adapted to test biocides for the aquaculture sector under conditions simulating practical use. The tested compound was suitable for NNV inactivation under at least some of the used conditions, which were critical in modulating the disinfectant’s effectiveness. For these reasons, disinfectant protocols need to be implemented with procedures such as cleaning and drying that lead to the best viral inactivation.

## Data Availability

The data presented in this study are available from the corresponding author upon reasonable request.
